# Solvent Influence on the Magnetization and Phase of Fe-Ni Alloy Nanoparticles Generated by Laser Ablation in Liquids

**DOI:** 10.3390/nano13020227

**Published:** 2023-01-04

**Authors:** Inna Y. Khairani, Qiyuan Lin, Joachim Landers, Soma Salamon, Carlos Doñate-Buendía, Evguenia Karapetrova, Heiko Wende, Giovanni Zangari, Bilal Gökce

**Affiliations:** 1 Chair of Materials Science and Additive Manufacturing, School of Mechanical Engineering and Safety Engineering, University of Wuppertal, 42119 Wuppertal, Germany; 2 Department of Materials Science and Engineering, University of Virginia, Charlottesville, VA 22903, USA; 3 Faculty of Physics and Center for Nanointegration Duisburg-Essen (CENIDE), University of Duisburg-Essen, 47057 Duisburg, Germany; 4 Advanced Photon Source, Argonne National Laboratory, Argonne, IL 60439, USA

**Keywords:** iron-nickel alloy, core-shell nanoalloys, nickel ferrite, hexagonal closed packed phase, carbon shell, laser synthesis of colloids

## Abstract

The synthesis of bimetallic iron-nickel nanoparticles with control over the synthesized phases, particle size, surface chemistry, and oxidation level remains a challenge that limits the application of these nanoparticles. Pulsed laser ablation in liquid allows the properties tuning of the generated nanoparticles by changing the ablation solvent. Organic solvents such as acetone can minimize nanoparticle oxidation. Yet, economical laboratory and technical grade solvents that allow cost-effective production of FeNi nanoparticles contain water impurities, which are a potential source of oxidation. Here, we investigated the influence of water impurities in acetone on the properties of FeNi nanoparticles generated by pulsed laser ablation in liquids. To remove water impurities and produce “dried acetone”, cost-effective and reusable molecular sieves (3 Å) are employed. The results show that the Fe_50_Ni_50_ nanoparticles’ properties are influenced by the water content of the solvent. The metastable HCP FeNi phase is found in NPs prepared in acetone, while only the FCC phase is observed in NPs formed in water. Mössbauer spectroscopy revealed that the FeNi nanoparticles oxidation in dried acetone is reduced by 8% compared to acetone. The high-field magnetization of Fe_50_Ni_50_ nanoparticles in water is the highest, 68 Am^2^/kg, followed by the nanoparticles obtained after ablation in acetone without water impurities, 59 Am^2^/kg, and acetone, 52 Am^2^/kg. The core-shell structures formed in these three liquids are also distinctive, demonstrating that a core-shell structure with an outer oxide layer is formed in water, while carbon external layers are obtained in acetone without water impurity. The results confirm that the size, structure, phase, and oxidation of FeNi nanoparticles produced by pulsed laser ablation in liquids can be modified by changing the solvent or just reducing the water impurities in the organic solvent.

## 1. Introduction

Iron nickel alloys are one of the most studied magnetic materials due to the abundance of their constituting elements on Earth [1,2] and owing to the interesting properties exhibited depending on their atomic ratio. For example, Invar (Fe_64_Ni_36_) exhibits very little thermal expansion (almost zero) over a wide temperature variation, while Permalloy (Fe_20_Ni_80_) offers a notably high magnetic permeability, low coercivity, and small magnetostriction [3,4,5]. Due to these interesting properties, iron-nickel alloys are employed in various key technologies such as transformers [6], magnetic actuators [7], magnetic sensors [8], electromagnetic shielding [9], spintronics [4,10], and catalysis [11,12,13]. The equiatomic iron-nickel alloy (Fe_50_Ni_50_), in particular, gained popularity as an electrocatalyst for the oxygen evolution reaction (OER) [12,14] and as a potential candidate for a permanent magnet after the discovery of the high-coercive tetrataenite mineral with L1_0_ structure found in a meteorite [2,15]. In both cases, the high material abundance of the alloy constituent elements on Earth represents a fundamental advantage, envisioned to overcome the price and supply chain problems associated with the current rare-earth-based OER catalysts (RuO_2_ and IrO_2_) and permanent magnets (NdFeB) in strategic technologies, such as electric mobility and energy storage. Other than the atomic ratio, the iron-nickel alloy particle size is also a crucial parameter defining their properties and performance in certain applications, especially for the catalytic activity of Fe_50_Ni_50_ alloy in the OER process [13], where nanosized materials are desired. By reducing the size to the nanometer range, especially below 10 nm, the specific surface area of the Fe_50_Ni_50_ catalyst is significantly increased, thus exposing more of its active sites for the reactions to take place. In addition, the recent report on the non-cubic symmetry in Fe_50_Ni_50_ nanoparticles [16] sparks the possibility of employing nanosized Fe_50_Ni_50_ as a rare-earth-free alternative to permanent magnets. These findings highlight the relevance of understanding and controlling the formation of Fe_50_Ni_50_ nanoparticles and explore novel synthesis techniques that allow Fe_50_Ni_50_ nanoparticles’ phase control.

Conventional fabrication methods of Fe_50_Ni_50_ nanoparticles (NPs) include chemical reduction and gas condensation routes. The chemical reduction of the iron and nickel salts with hydrazine in the presence of polyvinylpyrrolidone (PVP) resulted in face-centered cubic (FCC) Fe_50_Ni_50_ with an average diameter of 29 nm [17], and 96 nm without PVP [18]. Gas condensation of iron-nickel alloy in a helium atmosphere was sought, but oxidation took place on the surface of the particle after exposure to oxygen, resulting in the formation of core-shell NPs with FeNi γ-phase and oxides of γ-Fe_2_O_3_ or Fe_3_O_4_. These two methods, unfortunately, do not follow the green chemistry principles [19] due to the multi-step processes and the use of hazardous materials and inert gases to reduce oxidation. Meanwhile, pulsed laser ablation in liquid (PLAL) offers a one-step method to produce NPs directly in the desired liquid and avoids the generation of by-products, hence removing purification steps and the generation of extra chemical waste [20]. This technique does not require high vacuum or temperature conditions, making it easily implementable and transferable to industrial environments [21]. PLAL is based on the ablation of the bulk target in the desired liquid providing the versatility to tune the laser parameters and the ablation liquid to influence the temperature, pressure, and surrounding media [22]. By changing the liquid employed for PLAL, properties such as the composition and phase of the produced NPs can be modified [23].

Another NP property that is influenced by the liquid employed in the PLAL is the NP oxidation. Due to their small size and large surface area, NPs are prone to oxidation upon exposure to oxygen or oxidizing agents. In the PLAL, it is reported that the NP oxidation is influenced by the redox activity of the target material [24] and the choice of the ablation liquid [23]. For example, almost 100% of the surface of laser-generated Ti in water is oxidized, while less than 5% of the gold surface is oxidized [24]. For the ablation liquid, complete or partial oxidation of the NPs is observed in the ablation of Ti and Mn in water, which results in the generation of TiO_2_ [25] and Mn_3_O_4_ [26] NPs, respectively. NP oxidation might be purposely performed in some contexts where oxide NPs are desired, such as the generation of TiO_2_ by ablating Ti target immersed in water. However, in the cases where oxidation needs to be avoided, organic solvents such as acetone are known to reduce the oxidation of laser-generated NPs [27]. However, the oxidation itself is caused by the exposure of NPs inside the cavitation bubble to the oxygen species generated from the breakdown of liquid or gas in the nearby vicinity of the ablation spot; hence, all species with oxygen atoms might contribute to the oxidation of the NPs, including dissolved oxygen gas. To investigate this matter, Marzun et al. [28] ablated Cu in different ablation liquids, i.e., H_2_O (H_2_O_air_), H_2_O purged with Ar gas (H_2_O_ar_), and acetone. They reported the formation of Cu oxides in both H_2_O_air_ and H_2_O_ar_, with H_2_O_air_ having a higher oxidation level and amorphous phase in acetone. This indicates that not only the dissolved oxygen gases in the water contribute to the Cu oxidation, but also the water itself. Meanwhile, acetone with technical grade or laboratory grade, even with ACS reagent and HPLC grades, still contains water impurity to some extent (≤0.5%) [29,30], which contributes to the oxidation of the produced NPs.

In this study, the influence of water removal in acetone using molecular sieves on the oxidation level of the laser-generated Fe_50_Ni_50_ NPs is investigated. A molecular sieve is an adsorbent with three-dimensional frameworks of alumina-silicate, which is capable of reducing the water content down to 0.001% [31,32]. The molecular sieves provide an inexpensive and easy way to remove water from acetone, complying with the green chemistry principle due to its reusability. The removal of water in the organic solvents is not only intended to reduce the oxidation but also to directly encapsulate the NPs in a carbon shell during the PLAL synthesis that enhances their catalytic activity [33,34]. In addition to reducing the oxidation level and altering the shell formation, the generation of the non-cubic metastable hexagonal closed packed (HCP) in the organic solvents was investigated. It has been proposed that non-cubic phases might be used as a precursor to generating FeNi with L1_0_ structure [35], but the suggested methods to fabricate the non-cubic FeNi involve the use of high-pressure and high-temperature conditions such as in the diamond anvil cell (DAC) [36] or high-strain process [35]. Here, we propose PLAL as a method to produce the non-cubic HCP FeNi phase at room conditions, taking advantage of the locally high-pressure and -temperature conditions achieved by the high-intensity laser interaction with the target surface and surrounding liquid.

## 2. Materials and Methods

### 2.1. Fe_50_Ni_50_ Colloidal Nanoparticles Production

**Figure 1:**
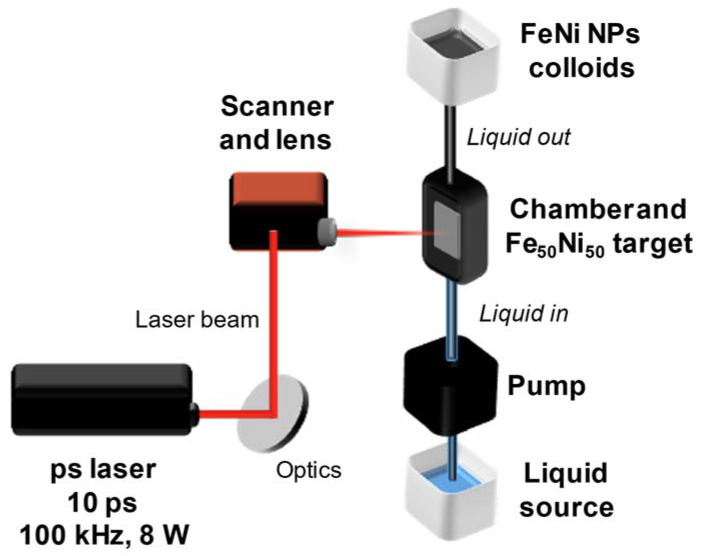
Schematic illustration of pulsed laser ablation in liquid using a flow chamber. The selected liquid flowed through the ablation chamber from the bottom to the top by a pump while the ablation took place. A Fe_50_Ni_50_ target was placed perpendicular to the incoming laser beam.

A picosecond laser Nd:YAG with a wavelength of 1064 nm, a pulse duration of 10 ps, a power of 8 W, a repetition rate of 100 kHz, a raw beam diameter of 2 mm, and a pulse energy of 80 µJ was employed to produce nanoparticles by PLAL (Figure 1). The laser beam was focused on the immersed (6 mm liquid layer) equiatomic FeNi alloy target by a galvanometric scanner coupled with an f-theta lens (focal length of 100 mm) following an Archimedean spiral pattern (6 mm diameter) with a speed of 2 m/s. The beam radius and peak fluence at the processing plane were 65 µm and 1.2 J/cm^2^, respectively. To avoid shielding of the laser beam by the produced nanoparticles, the liquid was pumped by a peristaltic pump at a flow rate of 150 mL/min (calibrated before the experiments of each liquid). The investigated liquids are distilled water, acetone, and “dried” acetone (obtained by immersing molecular sieves type 3 Å for 24 h to capture water molecules in acetone). The FeNi samples ablated in different liquids will be further referred to as FeNi in water, FeNi in acetone, and FeNi in dried acetone, respectively. All colloids have a similar absorbance value at the laser wavelength as shown in Figure S1. To dry the colloids and obtain nanopowders suitable for characterization, we performed magnetic separation using a permanent magnet (NdFeB) followed by liquid evaporation using an exhaust fan.

### 2.2. Analytical Methods

The generated FeNi NPs colloids were analyzed by transmission electron microscopy (TEM, JEOL JEM-2200FS, 200 kV, ZrO_2_/W emitter) and energy dispersive X-ray spectroscopy (EDX) to determine the size distribution, morphology, elemental composition, and oxide formation of the NPs. The sample was drop-casted on a copper grid with lacey carbon coating and was measured within one week after production to ensure minimum particle growth and oxidation due to aging.

Synchrotron X-ray diffraction (SXRD) was used to analyze the phase of the produced NPs qualitatively (using the peak matching technique) and quantitively (using the Rietveld refinement technique). Measurements were carried out at the 33-BM-C beamline of the Advanced Photon Source (APS) at the Argonne National Laboratory, United States with a beam wavelength of 0.77 Å. Since a wavelength of 0.77 Å was employed, the 2θ value is shifted to a lower degree compared to the standard 1.54 Å wavelength. The measurements were performed using the transmission (i.e., Debye-Scherrer) geometry. The colloids were sealed in Special Glass 10 capillaries (Hampton Research Corp.) by Beeswax (Hampton Research Corp.). The NPs generated in water were transferred to dried acetone to mitigate post-synthesis particle aging (i.e., oxidation and particle growth) before being loaded into capillaries for SXRD measurements.

Mössbauer spectroscopy was employed to determine the oxidation level and magnetic structure of the FeNi samples in acetone and dried acetone. Spectra of both powder and colloid samples were recorded in transmission geometry, with the latter being placed in an airtight sample container of appropriate geometry. A ^57^Co(Rh) radiation source was used, mounted on a constant-acceleration driving unit (WissEl GmbH), with low temperatures down to 4.3 K being achieved via a closed-cycle cryostat (Lake Shore Cryotronics). Spectra measured in external magnetic fields up to 8 T were recorded using a magnet cryostat (Oxford Instruments). Subspectra of magnetically ordered phases have been reproduced using hyperfine field distributions; isomer shifts are given relative to α-Fe at room temperature.

Nanoparticle magnetic behavior was studied using vibrating sample magnetometry (PPMS DynaCool, Quantum Design). Field-dependent magnetization loops M(H) were recorded at temperatures between 4.3–300 K and a magnetic field range of ±9 T.

## 3. Results and Discussion

### 3.1. Crystallographic Phases

**Figure 2:**
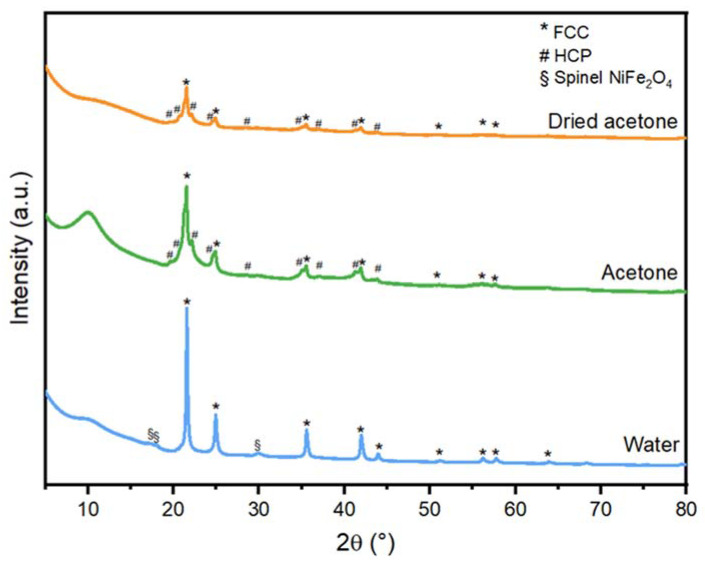
Synchrotron XRD profiles of the FeNi NPs ablated in different liquids. The ablation in acetone or dried acetone produced the FCC phase and the HCP phase, with a small volume of spinel NiFe_2_O_4_ phases (barely visible in this graph). Meanwhile, the ablation in water produced the FCC phase and NiFe_2_O_4_ phase. The complete indexing is presented in the supplementary information (Figure S2).

To determine the influence of different liquids on the phase formation of FeNi NPs, XRD phase analysis was performed. The SXRD profiles of FeNi NPs in dried acetone, acetone, and water are presented in Figure 2, and the complete indexing can be found in the supplementary (Figure S2). The FeNi NPs generated in water (Figure 2) show the diffraction peaks of the face-centered cubic (FCC) FeNi and the spinel NiFe_2_O_4_ structure. Meanwhile, the FeNi NPs generated in acetone and dried acetone (Figure 2) consist of the hexagonal closed-packed (HCP) FeNi phase in addition to the FCC FeNi phases and the spinel NiFe_2_O_4_ phases. To quantify the weight fraction (wt%) of the HCP phase, Rietveld refinement was performed (Figure S3) and the results are presented in Table 1.

All peaks corresponding to FCC and HCP phases were taken into account, while the NiFe_2_O_4_ peaks are excluded since the contribution of crystalline oxides in the XRD results is significantly low compared to the other phases. The NPs in dried acetone consist of 35.2 ± 1.0 wt% of the HCP phase, while the NPs in acetone account for 38.4 ± 0.2 wt% of the HCP phase, and the NPs in water contain no HCP phase (Table 1). It is interesting to observe that the ablation in acetone and dried acetone produces a mixture of the metastable HCP and stable FCC phases in the NP core, while only the FCC phase was formed in water. Meta- and stable phase mixtures in NPs produced by PLAL of different targets were previously reported. The formation of metastable zinc-blende and the stable diamond structures in silicon NPs [37], metastable hexagonal and stable cubic structures in diamond nanocrystals [38], the metastable γ-Fe and stable cubic FeO and α-Fe phases [39], and Ni NPs with stable FCC and metastable HCP phases [40,41]. Different arguments were postulated regarding the formation of stable and metastable phases during PLAL. (i) The specific heat capacity of the solvent, which influences the cooling rate of the ablation plasma plume generated during PLAL [40]. (ii) Shorter cavitation bubble lifetime compared to the theoretical lifetime according to the Rayleigh–Plesset theory [41], and (iii) the confinement of the cavitation bubble by the surrounding liquid, which induces the high temperature, high pressure, and high density (HTHPHD) state and shorter quenching time of the plasma plume in the liquid [37]. The above-mentioned hypotheses all pointed to the freezing of the metastable phase during the cooling (quenching), which preserves the metastable phases.

For FeNi alloy, the formation of a metastable FeNi HCP phase is usually associated with high-pressure and high-temperature conditions, such as in the Earth’s core [42,43,44,45,46]. It has been produced synthetically using a diamond anvil cell (DAC) from the bulk FeNi with a face-centered cubic (FCC) phase [36,46,47], where the sample is placed in a tiny space (3–4 mm) between two diamonds, which are pressed to each other [48]. Laser ablation in liquid also provides a high-pressure and high-temperature state to the nuclei inside the cavitation bubble (CB) and its collapse [49]. The bubble pressure during laser ablation might provide a suitable environment for the formation of the HCP phase; however, this cannot be the sole reason since the ablation in water does not produce HCP phases. The cavitation bubble dynamic study from the Choi group also showed that the cavitation bubble size was larger for hexane and acetonitrile compared to water [41]. The larger cavitation bubble and longer lifetime indicate lower pressure inside the bubble, as formerly reported from the laser ablation of aluminum oxide in ethanol, water, and isopropanol [50]. Hence, the pressure difference due to the cavitation bubble geometry would favor the HCP formation in water; however, it is only observed in organic solvents. Consequently, the liquid composition seems to be a significant factor influencing the FeNi NP’s phase.

Based on the results in Table 1, we have observed that the HCP phase does not scale with the fraction of water content in the organic solvent, consequently, this factor can be ruled out. However, the fact that the ablation in acetone (and dried acetone) produced an HCP phase, while the ablation in water only provided the FCC phase, suggests that the carbon-based solvent plays a significant role in the HCP phase formation. During PLAL, the presence of carbon species in the cavitation bubble generated from the interaction of the high-intensity laser with the organic solvent can influence the nucleation kinetics of the HCP and FCC phases. Hence, not only the FCC phase forms but also the HCP phase. When the cavitation bubble finally collapses, the fast temperature quenching freezes this metastable phase. Nevertheless, many factors related to the liquid and the laser ablation dynamics might form a complex system that contributes to the formation of the HCP phase in the FeNi NPs.

### 3.2. Oxide Formation

Oxidation of NPs, either partially or completely, changes the NP properties such as catalytic activity [51] and magnetization [52]. Controlling the oxidation level of laser-generated NPs is therefore important to produce NPs with the desired functionality. In this section, the influence of water impurity in acetone on the oxidation of laser-generated FeNi NPs is studied. Based on the XRD results (Figure 2), formations of minor amounts of spinel iron-nickel oxide NiFe_2_O_4_ (ICSD No. 241661) are observed in all studied samples, which shows that oxidation occurs even in dried acetone where most of the water molecules are captured by molecular sieves. Nevertheless, the amount of crystalline oxides in all of the samples is significantly low, approximately 0.7 wt% for FeNi in water, while for FeNi synthesized in acetone and dried acetone, the quantities are lower than the quantification error of the measurement/device, hence, the values are not of significance. Based on the study by Marzun et al., the ablation of a Cu target in water with an inert Ar atmosphere still resulted in oxidized species, due to the splitting of water molecules to reactive OH species. To avoid water impurities in acetone, we used molecular sieves. It was formerly reported that using the molecular sieve with the size of 4 Å for 21 h reduced the water content from 0.45% to 0.001% (*w*/*w*) [31]. Meanwhile, the water molecule has a diameter of 2.8 Å, hence, molecular sieves with a pore size of 3 Å were used to capture the water molecules in acetone and produce the “dried acetone”. Nadarajah et al. have investigated the influence of 3 Å molecular sieves to capture water molecules in acetone and reduce the oxidation level of the laser-ablated FeRh NPs. They reported that the use of molecular sieves resulted in less nanoparticle oxidation compared to NPs produced in untreated acetone [53] and they suggest that the bound oxygen atoms in acetone contribute to NP oxidation. The dissolved oxygen gas in the liquid is also found to partially oxidize NPs due to aging [28], which means that the oxidation occurs also due to the possibly prolonged NPs storage in the liquid before the analysis. Hence, the surface oxidation of FeNi NPs into spinel NiFe_2_O_4_ was likely caused by the NPs’ exposure to the oxygen species generated from the pyrolysis of the ablation liquid and later followed by the dissolved gas due to aging.

### 3.3. Morphology and Particle Size Distribution

**Figure 3:**
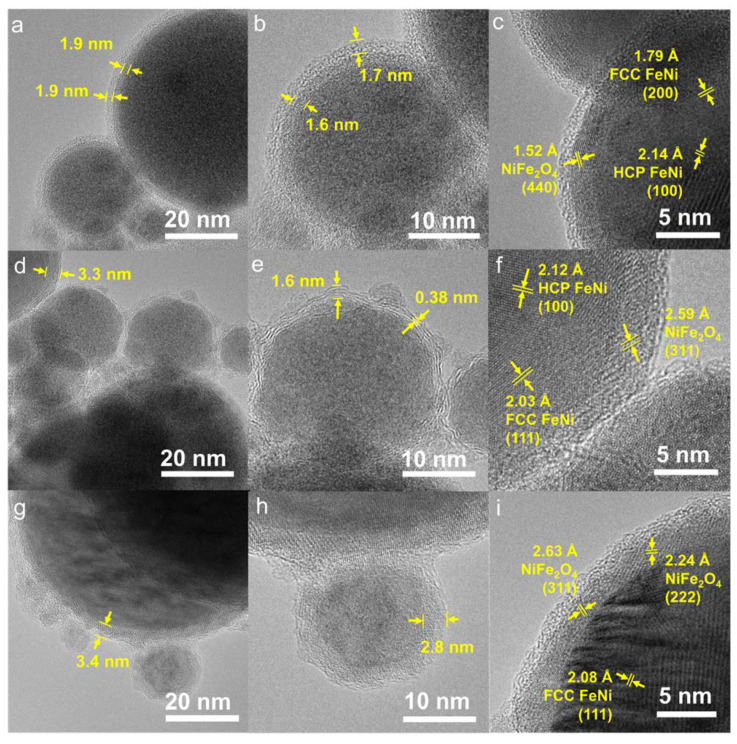
Morphology of FeNi NPs ablated in dried acetone (**a**–**c**), acetone (**d**–**f**), and water (**g**–**i**). (**a**,**d**,**g**) show the core-shell structure and the shell thickness of larger NPs, while (**b**,**e**,**h**) represent the smaller NPs. (**c**,**f**,**i**) confirm the phases observed in the XRD by measuring the lattice distance between the core and the shell.

The morphologies of the NPs ablated in dried acetone, water, and acetone are presented in Figure 3. Based on the bright field images of NPs in dried acetone (Figure 3a–c), core-shell structures with a core and two layers are formed, independently of the particle size. The thickness of the first layer (inner shell) ranges from 1.5 to 2.9 nm and has an average of 1.9 nm, whereas the average thickness of the second layer (outmost shell) was measured to be 2.4 nm, with a size range of 1.1–4.2 nm (Table 2). The core part shows a darker contrast in comparison to the shell, which can be explained as the change of electron scattering due to the electron density. The high electron density of the core part can be associated with the high material density, and in our case, it is Fe_50_Ni_50_ with a density of approximately 8.4 g/cm^3^. For the inner shell, the formation of a carbide or oxide layer is likely, as the ablation was performed in a solvent with molecularly bound carbon and oxygen atoms. The density of iron and nickel carbide are approximately 4.93 and 7.99 g/cm^3^, respectively, while iron, nickel, and iron-nickel oxide densities are between 5–7 g/cm^3^, which explains the lower contrast of the inner shell compared to the core. The formation of iron and nickel carbides and oxides after the ablation of Ni_50_Fe_50_ in acetone was previously reported, but there were still unidentified peaks around 52°, 71°, and between 75–90° despite efforts from the authors [33], which are identified as FCC and HCP peaks of FeNi in this study (Figure 2). XRD results in Figure 2 and the lattice distance observed in Figure 3c confirm that the inner shell of this sample is constituted by spinel iron-nickel oxide (NiFe_2_O_4_). Meanwhile, the outmost layer with the brightest contrast can be attributed to a carbon layer, which was formed due to the pyrolysis of organic solvent by the high-intensity pulses [27]. The laser radiation pyrolyzes the organic solvent and yields carbon species [54], which then become the building block of the outer NP layer. A small part of graphitic carbon is observed in this sample (Figure S4), but most of the observed carbon layers are amorphous.

Contrarily to the FeNi NPs in dried acetone, which exhibit the same core-shell structure for both small and large nanoparticle sizes, the sample in acetone (Figure 3) has two types of core-shell structures. Large NPs (d > 50 nm) form a core-shell structure, and the small NPs (d~20 nm) lean towards the formation of a core with outer graphitic carbon layers. The formation of a graphitic carbon layer was formerly reported after PLAL of metal targets in organic solvents, where the metal acts as a catalyst for the graphitization of the pyrolyzed carbon-based solvent [27,33]. Regarding the ablation of FeNi NPs in water, the formation of a core and a single shell structure for all NPs sizes was found. The formation of a single layer (without the carbon layer) is expected as water decomposes to H_2_ and O_2_ [54]. Based on the standard reduction potential, O_2_ acts as an oxidizing agent in the reaction with Fe and Ni, hence, the shell is most likely to be composed of oxides as supported by the XRD data (Figure 2).

**Figure 4:**
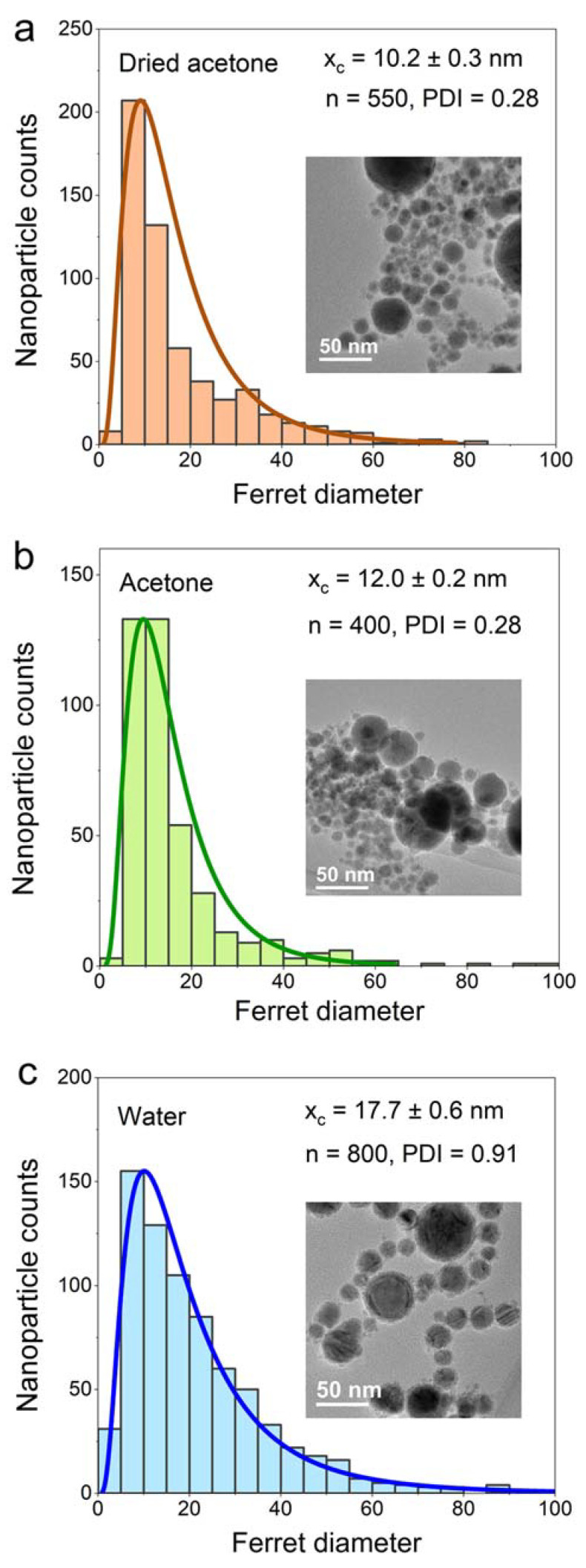
Number-weighted particle size distribution of FeNi NPs in (**a**) dried acetone, (**b**) acetone, and (**c**) water. FeNi NPs produced in dried acetone have the smallest median size and PDI, followed by FeNi produced in acetone, and FeNi produced in water. The number of counted particles (n) is denoted in the figures.

The particle size distribution of each sample was measured for at least 400 particles (Figure 4). All the histograms of the particle size distribution fit the log-normal distribution, which is common in PLAL-produced NPs. Meanwhile, the NPs produced through chemical synthesis methods, such as coprecipitation, hydrothermal, and sol-gel methods, usually have a Gaussian-type size distribution [18,55,56,57]. The average particle size of the sample is defined based on the center value of the log-normal fitting curve (x_c_) and the polydispersity index (PDI) is calculated from the square of standard deviation divided by the square of the mean value (σ^2^/µ^2^). The PDI is used to define whether the NPs are monodisperse or polydisperse, where a value of less than 0.3 is considered monodisperse [58]. The NPs size in dried acetone shows the lowest average particle size (x_c_) of 10.2 ± 0.3 nm, followed by NPs in acetone (12.0 ± 0.2 nm), and NPs in water (17.7 ± 0.6 nm). The PDI values of NPs in dried acetone, acetone and water are found to be 0.28, 0.28, and 0.91, respectively. Based on these results, the FeNi NPs in dried acetone and acetone can be considered monodisperse, while the FeNi NPs produced in water are polydisperse. The FeNi NPs in dried acetone and acetone are smaller than the FeNi in water due to the carbon coating on the NPs surface, which prevent the growth and coalescence of the NPs [59]. Nevertheless, it should be noted that further growth during storage cannot be completely ruled out even with carbon coating [60].

### 3.4. Elemental Composition

**Figure 5:**
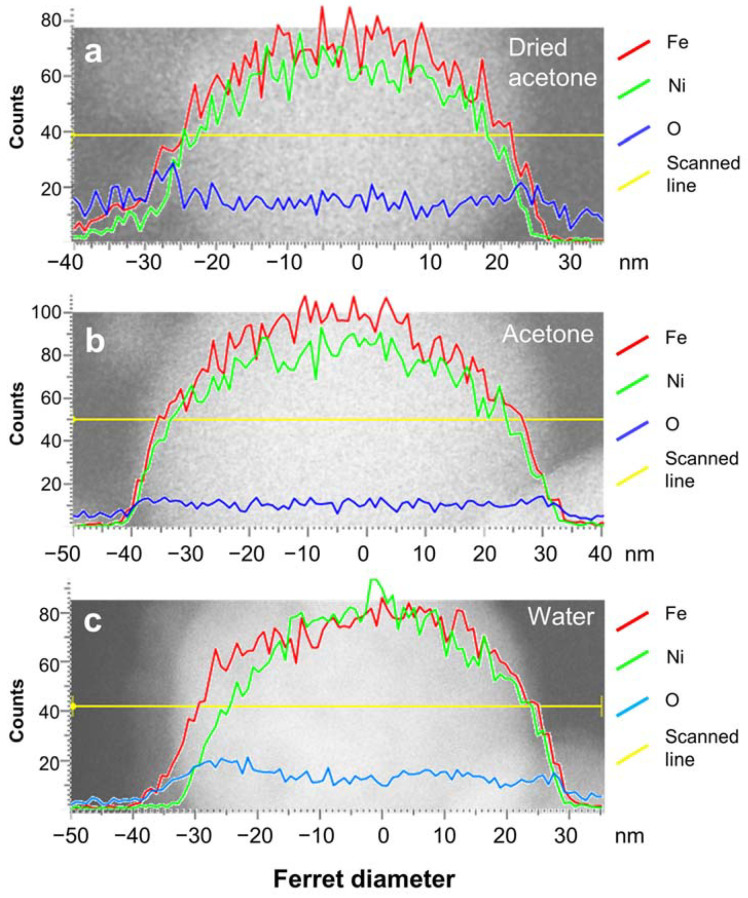
EDX-line scans of FeNi NPs ablated in (**a**) dried acetone, (**b**) acetone, and (**c**) water. The constant oxygen signals, which do not follow the Fe and Ni signals, indicate that oxidation only occurs on the NP surface.

To determine the elemental composition of the NPs’ core and shell, elemental scans using EDX-TEM were performed (Figure 5). A particle size of around 50 nm was selected as representative since the NPs generally have a distinct core-shell structure. Smaller NPs (10–20 nm) show distinct core-shell structures as well, but the oxygen signal from the environment sometimes provides a stronger contribution than the actual oxygen level on the NPs, hindering the oxidation analysis of the shell part (Figure S5). Hence, the discussion related to the EDX line scanning is limited to the larger NPs with a diameter of approximately 50 nm.

The EDX line scans (Figure 5) show that the Fe intensity is generally higher than the Ni intensity on the nanoparticle’s surface. This signal difference between Ni and Fe represents the composition of the shell, where Fe is present in a higher percentage compared to Ni. The oxygen intensity in all samples increases from the start of the shell where Fe is detected, then the value is constant throughout the particle. This shows that oxidation only occurs on the surface of the particle, but not in the core, where the Fe_50_Ni_50_ composition of the initial target is preserved. By assuming that all O atomic % (at%) belongs to the shell with a composition of NiFe_2_O_4_, the approximate Fe and Ni at% in the core part were calculated, as shown in Table 3. Note that the drop-casting was not performed in a glovebox; hence, it is likely that some oxygen adsorbs to the grid during the sample preparation prior to the TEM analysis. In addition, the accuracy of the device is around 1 at%, which might influence the estimation of the core composition. The at% of Fe and Ni in the core part of dried acetone, acetone, and water samples show almost similar values with a difference of around 1–3 at%, which means that the bulk composition is maintained. Jakobi et al. argued that similar heat of evaporation and density of Pt and Ir during the ablation of Pt_91_Ir_9_ in acetone produced NPs with similar stoichiometry as the target material [61]. The heat of evaporation of Ni and Fe are 370.4 kJ/mol and 349.6 kJ/mol, while the densities are 8.9 g/cm^3^ and 7.9 g/cm^3^, respectively. These similar values of heat of evaporation and density (1.06 and 1.13-factor difference, respectively) induce the simultaneous evaporation and condensation of the FeNi NPs alloy, which preserves the target’s elemental ratio. However, the oxidation level of the sample in dried acetone showed an unexpectedly high O at% value, which is even higher than water and twice the value of the sample in acetone. We have also measured the elemental composition using the EDX map scanning, which represents a larger area covering a larger number of NPs and also different NP sizes. As shown in Figure S6 and Table S1, the O at% of the dried acetone sample is the lowest, with a 15 at% difference compared to the acetone sample. There is also an anomalous trend where O at% of the water sample is slightly lower by almost 3 at% compared to the acetone sample. Therefore, we believe that the measurement of O at% from EDX-TEM fails to provide a complete representative value for the whole sample and includes the contribution of all the NP sizes, leading to a variation of the O at% values obtained for different NPs or analyzed areas. Thus, we sought another measurement, i.e., Mössbauer spectroscopy, to define the oxidation level of the whole sample with higher statistical confidence.

**Figure 6:**
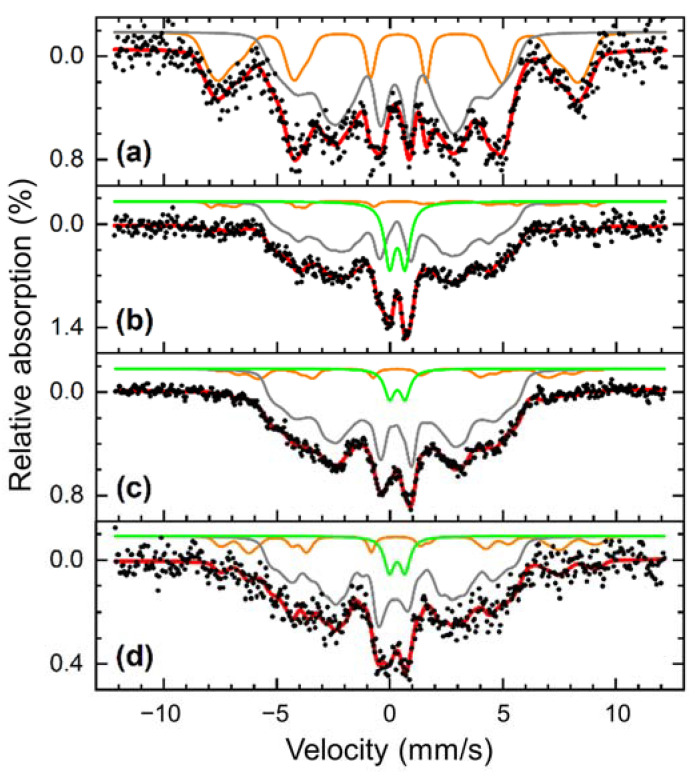
Mössbauer spectra of FeNi NP colloids: Prepared from dried acetone, aged for four months measured at 4.3 K (**a**) and 80 K (**b**), from dried acetone in the initial state at 80 K (**c**) and acetone in the initial state at 80 K (**d**). Spectra consist of an outer sextet distribution (orange) assigned to NiFe_2_O_4_, an inner sextet distribution corresponding to metallic FeNi (grey), and a doublet contribution (green) assigned to oxide material in the para- or superparamagnetic state.

Mössbauer spectroscopy was employed to quantify the total oxide fraction of the FeNi NPs and their aging behavior for longer oxidation times. The measurements were performed in transmission geometry, providing a measurement signal averaged over the total sample volume, thus, giving a comprehensive overview of the composition of Fe-bearing phases in the nanoparticles as well as their general magnetic structure. Due to different hyperfine interactions of the Fe nuclei with their surroundings, metallic and oxidic Fe-bearing phases result in distinctively different sub-spectra, as visible in Figure 6a. At ca. 4.3 K, two broadened sextet distributions can be identified for the aged, dried acetone sample: a larger one with moderate hyperfine magnetic fields B_hf_ and an average isomer shift of ca. 0.30 mm/s (green) assigned to metallic FeNi, and a second one with a larger B_hf_ and an isomer shift of ca. 0.47 mm/s. The latter is usually indicative of ferric oxides [62,63], whereby this distribution is assigned to iron atoms in the NiFe_2_O_4_ shell. Due to the very broad structure of the metallic FeNi subspectrum, a resolution of HCP- and FCC-components was not feasible.

Studying the spectrum at 80 K in Figure 6b in comparison, we observe only minor changes in the metallic FeNi subspectrum, while the oxidic sextet almost vanished, now manifesting mainly in a para- or superparamagnetic doublet state (green), both being mainly identical in spectral intensity and isomer shift. A more detailed analysis can be found in Figure S7, showing the dried acetone FeNi nanopowder spectra between 5 K and room temperature without an external magnetic field. This was done to study whether complete evaporation of the liquid to produce a powdered sample resulted in an oxidation increase due to the exposure to air, and the possibility of storing the colloids as a powder without influencing the oxidation level.

From the spectrum at 5 K, where the sub-spectra can be well resolved, 26% of the spectral area is assigned to the oxide fraction, which would suggest that further oxidation of this sample takes place following drying and storage before the measurement was completed. This proves our earlier point, that it is important to use freshly produced colloids without extended storage time, either in their original liquid or as dried NP powder. To further reduce the oxidation level, it is also possible to use an organic solvent with no molecularly bound oxygen, such as acetonitrile, or H_2_ as a reducing gas. However, the reduced price and the reusability of the molecular sieves employed in this work, which could be re-activated by heat treatment at around 300 °C, offer a beneficial option for the oxidation control of PLAL-generated NPs and the cost-effective upscaling of the production as required for catalysis applications. At higher temperatures, it is found that the sextet to doublet transition of the NiFe_2_O_4_ component mainly takes place between 30 and 60 K. No considerable changes in the spectral structure are visible above ca. 100 K. Corresponding measurements up to room temperature were not attainable for the colloidal samples since Brownian nanoparticle motion leads to severe line broadening, hindering a detailed analysis [64,65].

For the dried acetone colloid 4 months after production shown in Figure 6a,b, the NiFe_2_O_4_ sub-spectra contains roughly 27% of the spectral area. Assuming the oxide shell consists of stoichiometric NiFe_2_O_4_ based on the previous XRD results (Figure 2) and expecting similar Debye-Waller factors for metallic FeNi and NiFe_2_O_4_ at cryogenic temperatures, relative spectral areas represent a simple approximation of the weight percentage (wt%) of the corresponding phase due to very similar atomic Fe fractions per mass. To evaluate the effect of reducing water content on the total oxide fraction as well as the stability of the prepared nanoparticles, the oxide spectral area in aged, and dried acetone colloid is compared to fresh dried acetone (14%) and fresh acetone colloid (22%) shown in Figure 6c,d. The results clearly show a lower oxide fraction after preparation in mole-sieved acetone and minor ongoing oxidation upon a longer aging time. It can be concluded that while the drying process is effective, the reduced oxidation of the sample is lost again after extended storage time, and results in a similar oxide fraction as the fresh colloid made from the commercial, untreated acetone. This also means that the carbon shell and the NiFe_2_O_4_ shell on the NPs did not completely stop further oxidation of the NPs during longer storage time. Oxidation might occur from the presence of molecularly bound O atoms in acetone or the dissolved O_2_ gas. Therefore, it is important to use fresh colloids in the posterior intended catalysis or magnetic application to avoid further oxidation that can detriment the produced FeNi NPs performance.

### 3.5. Magnetic Properties

**Figure 7:**
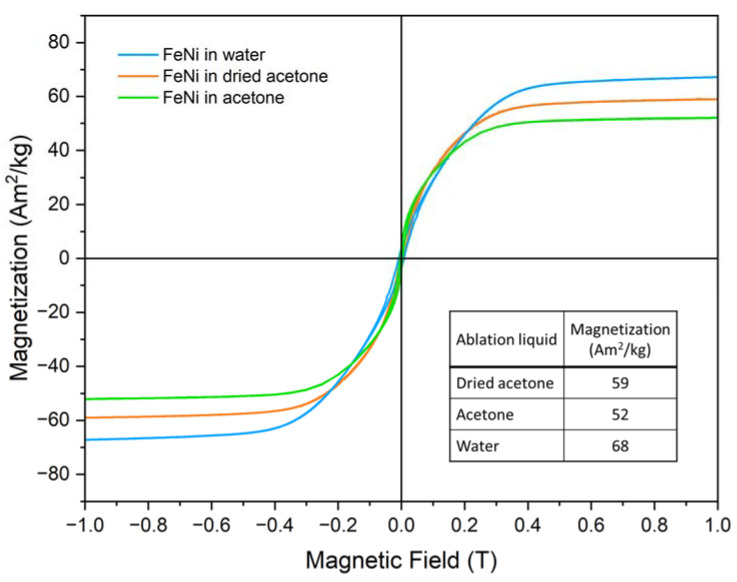
M(H) curves of FeNi nanoparticle powders from colloids prepared in different liquids at room temperature (300 K). FeNi in acetone data reproduced from [67].

The magnetic field-dependent magnetization M(H) curves of FeNi NPs formed in different liquids are shown in Figure 7, recorded at 300 K up to a maximum magnetic field of 1 T. A similar saturation alignment for the three samples can be observed, with the overall character of the M(H) curves being comparable, reaching high magnetization values already at ca. 0.4 T and showing a gradual further increase in the high-field region. Based on Mössbauer spectroscopy in-field experiments as shown in Figure S8, this M(H) shape can be explained as follows: A distinctively reduced intensity of lines 2 and 5 of the FeNi subspectrum can be observed already at a magnetic field of 1 T visible in Figure S8b, revealing a state of almost complete magnetic alignment for the metallic core of the nanoparticles [66]. The NiFe_2_O_4_ shell, on the other hand, displays high intensities of lines 2 and 5 even up to 8 T (Figure S8c), proving that magnetic moments here are still relatively random in their orientation, resulting in a limited oxide contribution to magnetization, slowly increasing when going to higher fields. The incomplete magnetic alignment of the oxide shell is also clearly evident by the only partial resolution of the contributions from A- and B-spinel lattice positions at 8 T.

Clear differences are apparent when regarding the 1 T magnetization values. When comparing the acetone and dried acetone samples, the effect of the drying process becomes clear, as the mole-sieved sample has a higher magnetization of ca. 59 Am^2^/kg compared to the 52 Am^2^/kg of the non-sieved sample, which can presumably be attributed to the lower oxidation of the former. However, the sample produced in water shows an even higher magnetization at 68 Am^2^/kg. It would be prudent to assume that this difference stems from the fact that the particle size of the water-based sample is significantly higher than that of the two acetone-based ones, which would lead to a lower surface-to-volume ratio and thus a reduced amount of surface spin canting. To discern this difference, additional magnetometry measurements were performed, up to higher fields of 9 T and in a wide range of temperatures from 4.3 K up to 300 K, as shown in Figure S9 for the dried acetone and water-based samples. Here, two aspects can be discussed: on the one hand, the low temperature, and high field measurements show that the water-based sample still retains a slightly higher magnetization value at 9 T of 82 Am^2^/kg compared to 76 Am^2^/kg for the dried acetone sample. Interestingly, the slightly more pronounced shape of the M(H) curves for the water-based sample indicates that full saturation has not yet been reached at 9 T, which would suggest that the higher magnetization value compared to the acetone-based samples is not solely due to a reduced occurrence of spin canting due to the larger average particle size. An explanation can be provided by the paramagnetic HCP phase being present in the acetone-based samples, but not in the water-based one, leading to a reduction of the overall measured magnetization. Additionally, the previously mentioned carbon shell formation can also contribute to this effect. However, despite this slight decrease relative to the water-based sample, the difference in magnetization visible between the sample formed in dried and regular acetone clearly shows the viability of the method presented here to prevent undesired oxidation of the FeNi NPs.

## 4. Conclusions

Reducing water impurities in acetone for the generation of Fe_50_Ni_50_ nanoalloys by PLAL influences the phases, core-shell structure, oxidation, and magnetic property of the produced NPs. FeNi NPs in dried acetone with reduced water impurity exhibit FCC and HCP phases in the core, an inner NiFe_2_O_4_ shell, and an outer amorphous carbon shell (FCC/HCP FeNi@NiFe_2_O_4_@amorphous carbon). The NPs in commercial, untreated acetone (water impurity of 0.3–0.5%) produced a mixture of FCC and HCP phases in the core with either NiFe_2_O_4_ shell or graphitic carbon (FCC/HCP FeNi@NiFe_2_O_4_ and FCC/HCP FeNi@graphitic carbon). Meanwhile, ablating FeNi alloy in water produced FCC core and NiFe_2_O_4_ shell (FCC FeNi@ NiFe_2_O_4_) NPs without any traces of the HCP phase or carbon shell. Reducing water impurity in acetone was found to lower the oxidation level by 8% (total oxide fraction, as measured by Mössbauer spectroscopy) compared to the NPs in untreated acetone. The magnetization of the dried acetone sample (59 Am^2^/kg) was higher than the acetone sample (52 Am^2^/kg) due to the lower degree of oxidation. The NPs produced in water exhibit a higher magnetization of 68 Am^2^/kg. The higher magnetization in the water sample is due to the larger average NPs size (17 nm), compared to the NPs in dried acetone (10 nm) and acetone (12 nm). The smaller average size of NPs in acetone-based liquid is related to the carbon layer formed in the ablation plume, which constrains the particle growth. The diverse core-shell structure and the modified FeNi NPs properties observed in this study show that FeNi NPs with different phase and shell structures can be generated just by reducing the amount of water impurity in the organic solvent or modifying the solvent employed in PLAL. This opens up a straightforward synthesis approach of different core-shell FeNi NPs that can be adapted to the broad fields where FeNi NPs are applied, such as sensors and actuators development, catalysis, magnetism, or biomedicine.

## Figures and Tables

**Table 1:** The crystalline phase composition of FeNi NPs synthesized in different ablation liquids. The weight fraction of the HCP FeNi phase was extracted from the SXRD profile by Rietveld refinement.

Ablation liquid	Crystalline Phase Composition	HCP Content (wt%) *
**Dried acetone**	FCC FeNi, HCP FeNi, NiFe_2_O_4_	35.2 ± 1.0
**Acetone**	FCC FeNi, HCP FeNi, NiFe_2_O_4_	38.4 ± 0.2
**Water**	FCC FeNi, NiFe_2_O_4_	0

* The HCP content reported here is the weight fraction of the FeNi HCP phase with respect to the weight of the nanoparticle core. The Rietveld refinement was performed on a structure model of the nanoparticle core that consisted of the FCC FeNi phase(s) and the HCP FeNi phase if existing. The NiFe_2_O_4_ phase in the particle shell was not included in the structure model being refined.

**Table 2:** FeNi NPs in dried acetone, acetone, and water shell thicknesses as obtained by TEM.

Ablation Liquid	Average Particle Size (x_c_, nm)	Core Phase	Shell Phase	Shell Thickness (nm)	
				Average (Mean)	Range (Min to Max)
Dried acetone	10.2 ± 0.3	HCP/FCC FeNi	NiFe_2_O_4_	2.4	1.1–4.2
			Amorphous carbon	1.9	1.5–2.9
Acetone	12.0 ± 0.2	HCP/FCC FeNi	NiFe_2_O_4_	2.3	1.4–3.5
			Graphitic carbon	1.2	0.7–1.9
Water	17.7 ± 0.6	FCC FeNi	NiFe_2_O_4_	4.9	2.4–9.8

**Table 3:** Elemental composition (in at%) of the NPs shown in Figure 5 and the estimation of Fe and Ni at% in the core part, assuming that all oxygen at% belongs to the NiFe_2_O_4_ shell.

Ablation Liquid	Whole Particle Composition			Shell Composition *			Core Composition **	
	Fe at%	Ni at%	O at%	Ni at%	Fe_2_ at%	O_4_ at%	Fe at%	Ni at%
Dried acetone	35.1	29.3	35.6	8.9	17.8	35.6	17.3	20.4
Acetone	43.6	38.1	18.2	4.6	9.1	18.2	34.5	33.6
Water	38.7	33.6	27.7	6.9	13.9	27.7	24.9	26.7

* with the assumption that all O at% of the particle comes from the NiFe_2_O_4_ shell, ** subtracting the whole particle composition with the shell composition.

## Data Availability

Besides the data published in this article, no new data were created.

## Supplementary Materials

The following supporting information can be downloaded at: https://www.mdpi.com/article/10.3390/nano13020227/s1, Figure S1: The normalized absorption spectra of Fe50Ni50 nanoparticles generated in dried acetone (black), acetone (red), and water (blue) within the wavelength of 400–1100 nm; Figure S2: Phase identification of FeNi NPs in dried acetone, acetone, and water based on the Synchrotron XRD results; Figure S3: Rietveld refinement of the synchrotron XRD results without the contribution of the oxide phase; Figure S4: HR-TEM image of NP in dried acetone which shows the formation of graphitic carbon; Figure S5: Line scanning EDX-TEM of small NP (d = 16 nm) of FeNi ablated in acetone (top) and in dried acetone (bottom); Figure S6: EDX map scanning of the FeNi NPs in different ablation liquids. Figure S7: Mössbauer spectra of the dried acetone colloid powder sample recorded between 5 K and room temperature. Figure S8: Mössbauer spectra of an aged dried acetone powder sample recorded at ca. 4.3 K in external magnetic fields of (a) 0 T, (b) 1 T, and (c) 8 T parallel to γ-ray incidence direction. Figure S9: Field-dependent magnetization of FeNi nanoparticle powder from the (a) dried acetone and (b) the water-based sample recorded at 4.3 K to 300 K in magnetic fields up to 9 T. Table S1: The elemental composition of the whole area (in at%) obtained by map scanning as shown in Figure S5 and the estimation of Fe and Ni at% in the core part, assuming that all O at% belongs to the NiFe_2_O_4_ shell. Reference [68] is cited in the supplementary materials.
