# Supplementary material for: Solvent Influence on the Magnetization and Phase of Fe-Ni Alloy Nanoparticles Generated by Laser Ablation in Liquids
Source: Nanomaterials (Basel). 2023 Jan 4;13(2):227. doi: 10.3390/nano13020227 (PMC9864041; doi:10.3390/nano13020227)
Supplement: Supplementary file 1 [file nanomaterials-13-00227-s001.zip › nanomaterials-2114288-supplementary.pdf]

# Supplementary Information

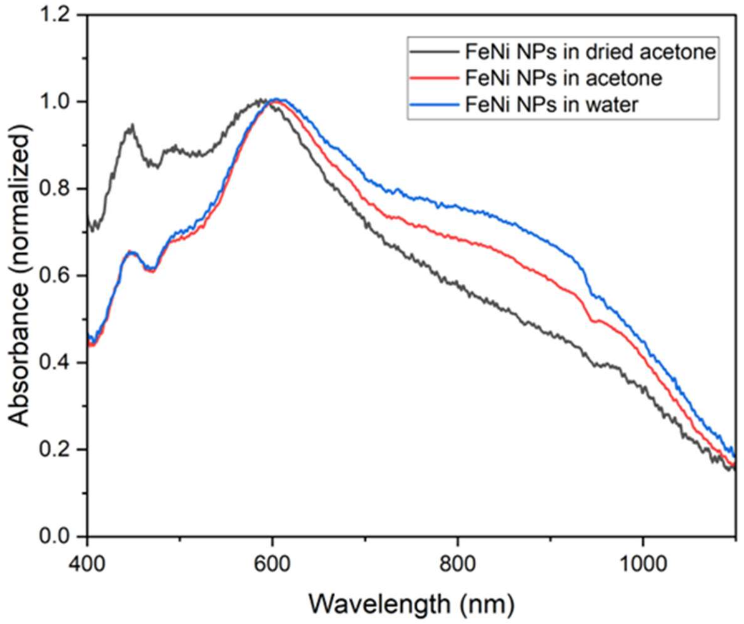


Figure S1. The normalized absorption spectra of $Fe_{50}Ni_{50}$ nanoparticles generated in dried acetone (black), acetone (red), and water (blue) within the wavelength of 400–1100 nm.

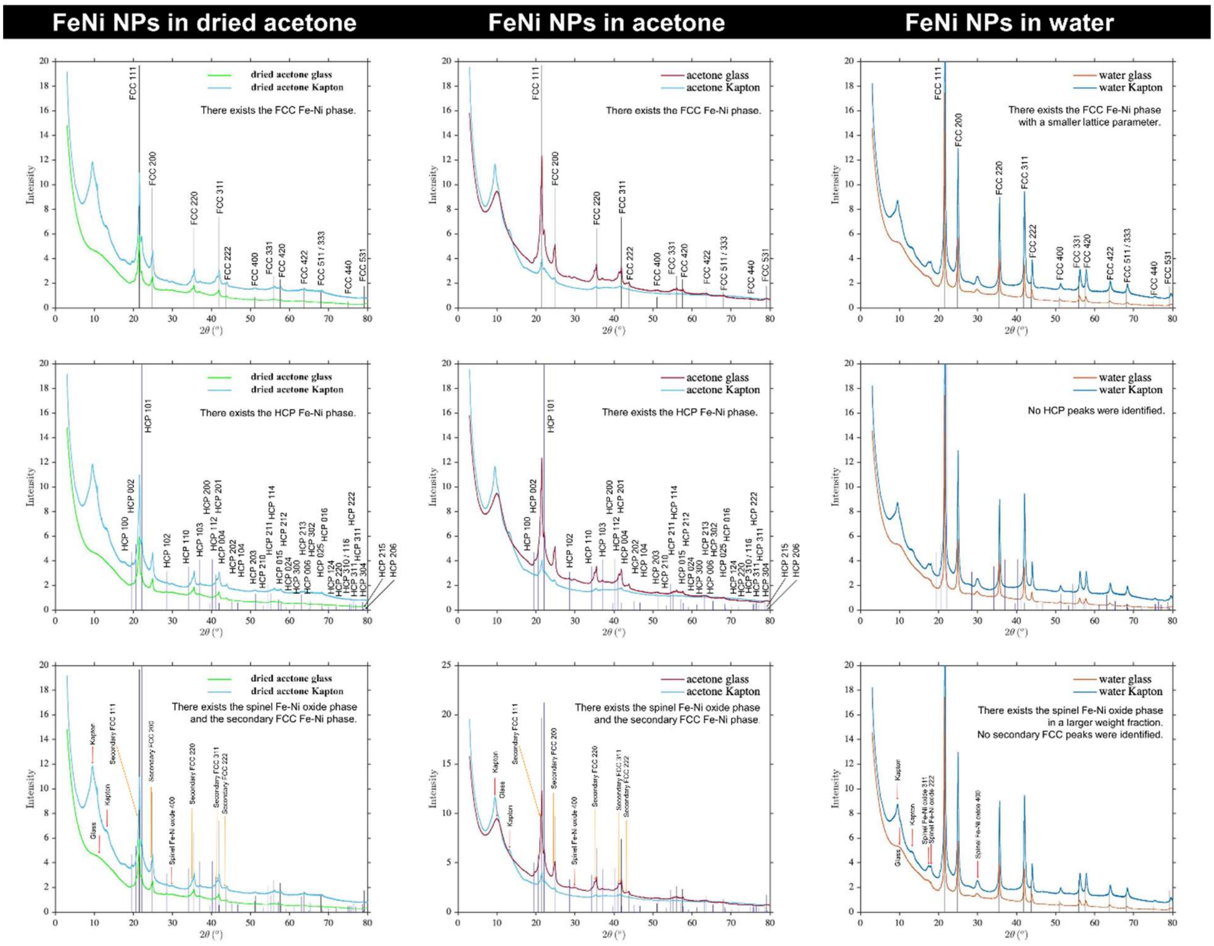


Figure S2. Phase identification of FeNi NPs in dried acetone, acetone, and water based on the Synchrotron XRD results. Note that all samples display spinel $NiFe_2O_4$ peaks and that the diffractogram FeNi NPs in water shows no sign of HCP peaks.

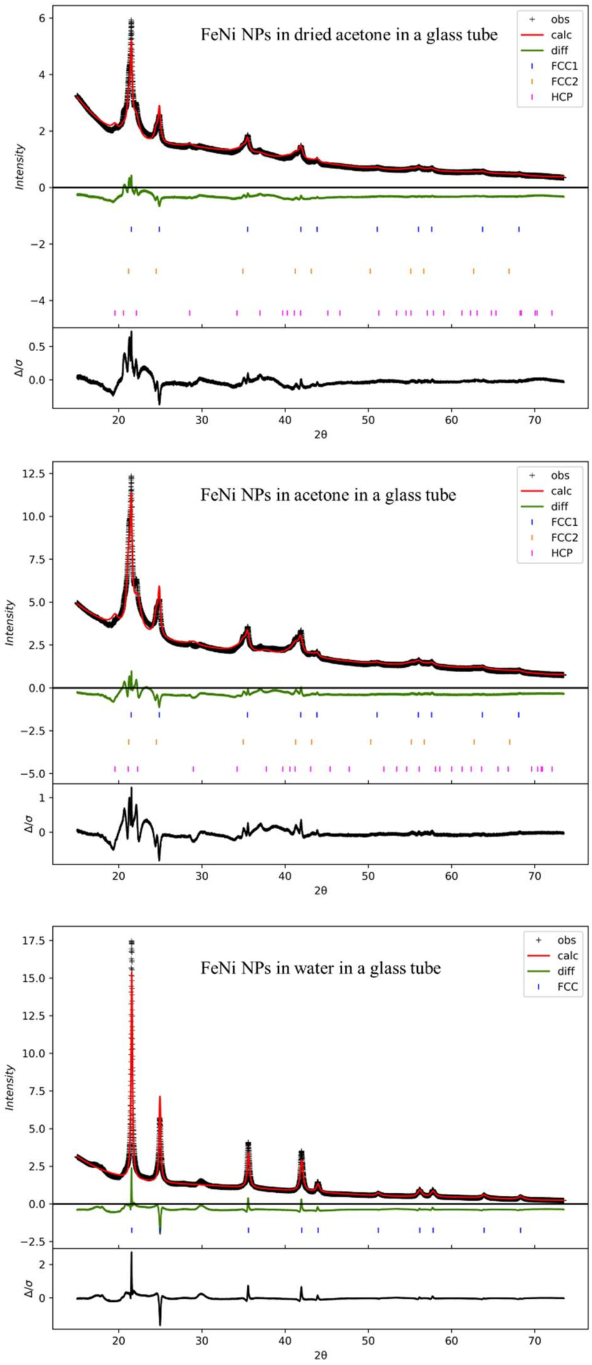


Figure S3. Rietveld refinement of the synchrotron XRD results without the contribution of the oxide phase. The crystalline phase of the oxide is very small (0.7 wt% for the FeNi NPs generated in water, and the quantities are lower than the quantification error of the device for the FeNi synthesized in acetone and dried acetone), hence, it can be neglected.

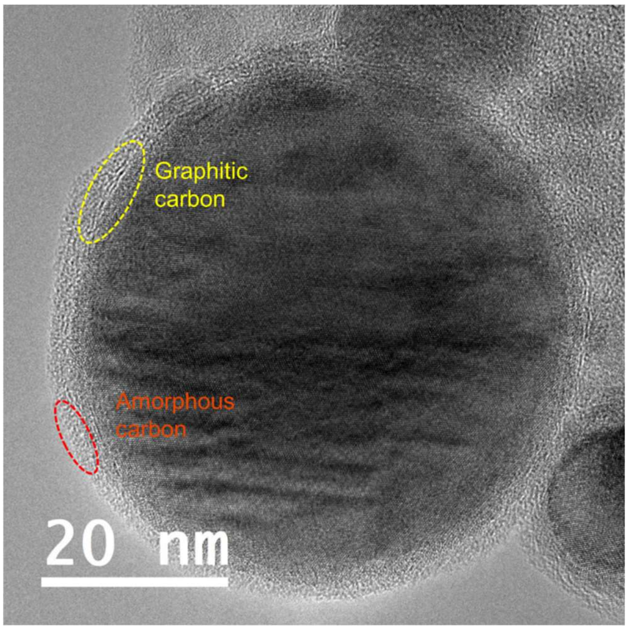


Figure S4. HR-TEM image of NP in dried acetone which shows the formation of graphitic carbon.

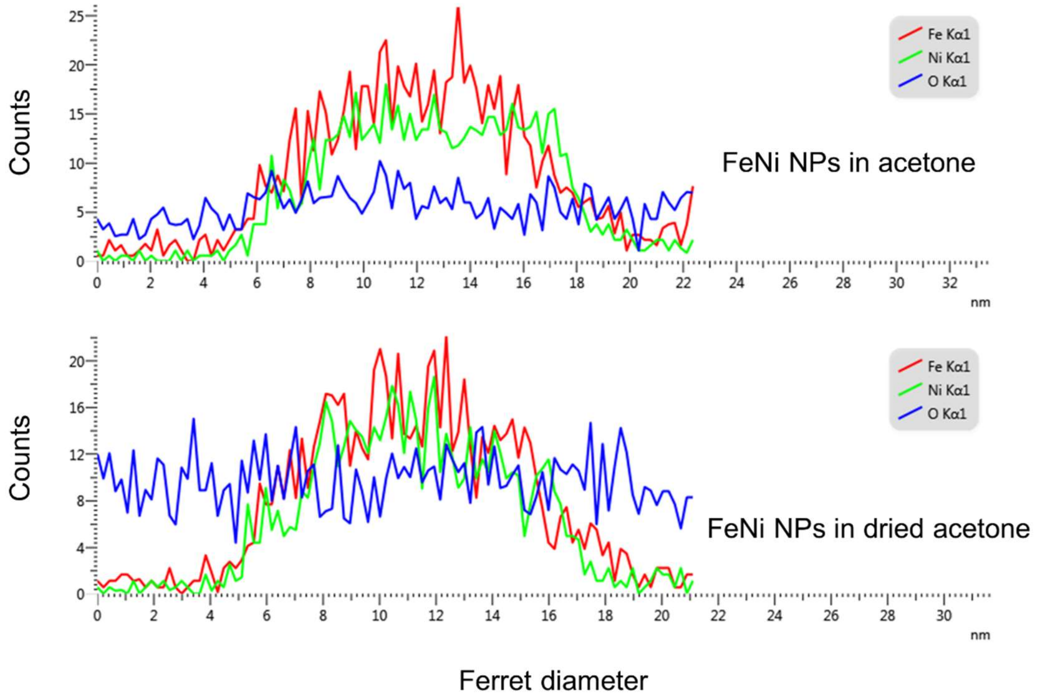


Figure S5. Line scanning EDX-TEM of small NP (d = 16 nm) of FeNi ablated in acetone (top) and in dried acetone (bottom). For the NP in dried acetone, the oxygen signal does not change with the height of the NP, which suggests that the detected oxygen atoms come from the surrounding environment instead of the particle.

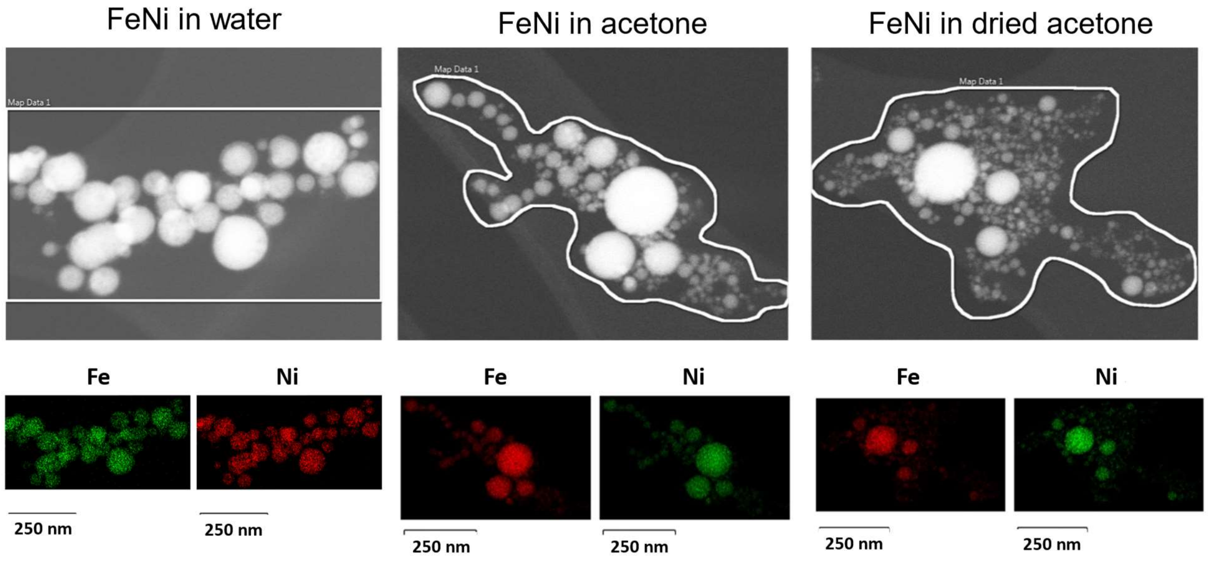


Figure S6. EDX map scanning of the FeNi NPs in different ablation liquids.

| **Ablation liquid** | **Whole area composition** | | | **Shell composition*** | | | **Core composition**** | |
|---|---|---|---|---|---|---|---|---|
| | **Fe at%** | **Ni at%** | **O at%** | **Ni at%** | **$Fe_2$ at%** | **$O_4$ at%** | **Fe at%** | **Ni at%** |
| **Dried acetone** | 42.4 | 35.5 | 22.1 | 5.5 | 11.1 | 22.1 | 31.4 | 30.0 |
| **Acetone** | 34.2 | 28.6 | 37.2 | 9.3 | 18.6 | 37.2 | 15.6 | 19.3 |
| **Water** | 37.2 | 28.6 | 34.3 | 8.6 | 17.2 | 34.3 | 20.1 | 20.0 |

**with the assumption that all O at% of the area comes from the $NiFe_2O_4$ shell*

***subtracting the whole area composition with the shell composition*

Table S1. The elemental composition of the whole area (in at%) obtained by map scanning as shown in Figure S6 and the estimation of Fe and Ni at% in the core part, assuming that all O at% belongs to the $NiFe_2O_4$ shell

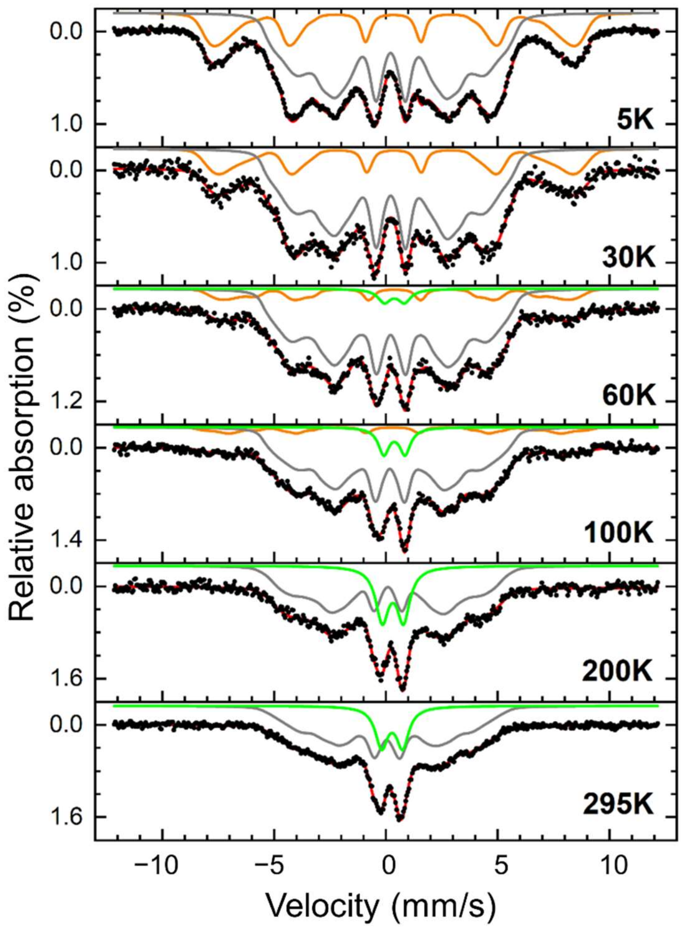


Figure S7. Mössbauer spectra of the dried acetone colloid powder sample recorded between 5 K and room temperature. Spectra consist of an outer sextet distribution (orange) assigned to $NiFe_2O_4$, an inner sextet distribution corresponding to metallic FeNi (grey), and a doublet contribution (green) assigned to oxide material in the para- or superparamagnetic state.

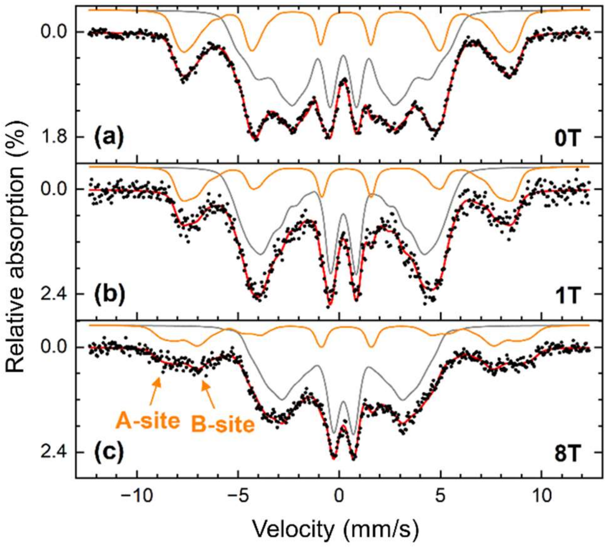


Figure S8. Mössbauer spectra of an aged dried acetone powder sample recorded at ca. 4.3 K in external magnetic fields of (a) 0 T, (b) 1 T and (c) 8 T parallel to γ-ray incidence direction. Arrows mark partially resolved contributions of spinel A- and B- (tetrahedral and octahedral) lattice positions.

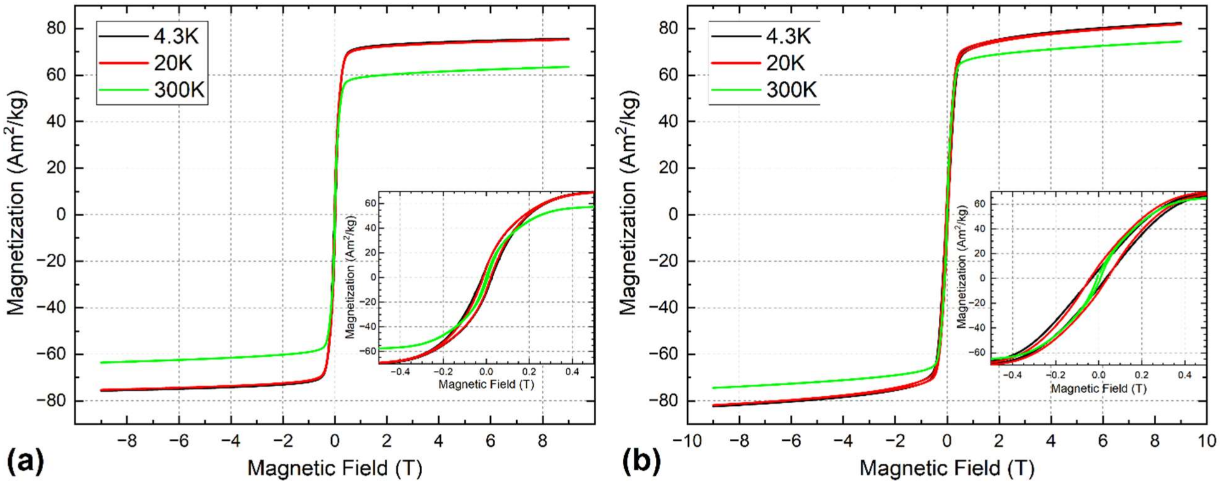


Figure S9. Field-dependent magnetization of FeNi nanoparticle powder from the (a) dried acetone and (b) the water-based sample recorded at 4.3 K to 300 K in magnetic fields up to 9 T. Insets show the low-field regions to determine coercive fields.

The Rietveld refinements of the SXRD patterns (shown in Figure S3) were performed using the GSAS-II software package [Toby 2013].

1. Le Bail Fitting of the SXRD Pattern of Standard $LaB_6$

The wavelength of the synchrotron radiation and the instrument parameters were extracted from the SXRD pattern of $LaB_6$ (SRM 660a) collected at APS 33-BM-C by the Le Bail fitting implemented in GSAS-II. The wavelength of the synchrotron radiation was extracted to be $\lambda$ = 0.775 Å. The instrument parameters extracted were: $U$ = -13.703; $V$ = 14.693; $W$ = -0.034; $X$ = -1.744; $Y$ = 2.501; $SH/L$ = 0.026. The histogram ranged refined were 9.0–79.0° 2θ. The background function was defined as a 6-term cosine function. The background parameters, histogram scale factor, instrument parameters, and wavelength were refined altogether in the last refinement calculation, resulting in the weighted $R$ factor of $wR$ = 14.36%. The wavelength and the instrument parameters were used as fixed parameters in the subsequent Rietveld refinement of the SXRD patterns of the Fe-Ni NPs collected on the same beamline.

2. Rietveld Fitting of the SXRD Pattern of the FeNi NPs Ablated in Water

The refinement of the SXRD pattern of the FeNi NPs ablated in acetone was also based on a structure model consisting of a single FCC phase with the lattice parameter of $a_{FCC}$ = 3.59 Å. The histogram range refined were 15.0–73.5° 2θ. To define the background, a set of fixed points were selected and an 8-term cosine function was used to fit the set of fixed points being selected. The parameters refined in the last refinement calculation include the histogram scale factor, the lattice parameter, and the microstrain, resulting in the weighted $R$ factor of $wR$ = 7.60%.

3. Rietveld Fitting of the SXRD Pattern of the FeNi NPs Ablated in Dried Acetone/Acetone

The refinement of the SXRD pattern of the FeNi NPs ablated in dried acetone/acetone was based on a structure model consisting of 3 phases: (1) a primary FCC phase with the lattice parameter of $a_{FCC}$ = 3.60 Å; (2) a secondary FCC phase with the lattice parameter of $a'_{FCC}$ = 3.65 Å; (3) a HCP phase with the lattice parameters of $a_{HCP}$ = 2.64 Å and $c_{HCP}$ = 4.33 Å. The histogram range refined were 15.0–73.5° 2θ. To define

the background, a set of fixed points were selected and an 8-term cosine function was used to fit the set of fixed points being selected. The parameters refined in the last refinement calculation include the lattice parameter(s), the weight fraction, and the microstrain of each of the 3 phases. To quantify the variation of these refinement restuls with respect to the selection of the fixed points for the background determination, the entire refinement routine was repeated 3 times, and the average and the standard deviation were reported. For example, the weight fraction reported to be 35.2 ± 1.0% was calculated from the 3 refined values (36.3%, 34.3%, and 35.0%) from the 3 repetitions, respectively. All the refinement repetitions resulted in a weighted $R$ factor of $wR = 5 \sim 6\%$.

References:


1. Toby, B. H., & Von Dreele, R. B. (2013). "GSAS-II: the genesis of a modern open-source all purpose crystallography software package". *Journal of Applied Crystallography*, **46**(2), 544-549. doi:10.1107/S0021889813003531
